# IL‐2 silencing enables Tfh cell expansion during vaccination but is redundant for antibody production

**DOI:** 10.1111/imcb.70084

**Published:** 2026-02-24

**Authors:** Silvia Innocentin, Ross McKenzie, Jayalini Assalaarachchi, Helena A Carslaw, Anusha Gupta, Sanne Cole, Adrian Liston, Michelle A Linterman, Louise Webb, Alice R Burton

**Affiliations:** ^1^ Lymphocyte Signaling and Development Babraham Institute Cambridge UK; ^2^ Department of Pathology University of Cambridge Cambridge UK

**Keywords:** Germinal center, IL‐2, T‐follicular helper, vaccination

## Abstract

Vaccines stimulate protective humoral immunity by coordinating lymphocyte activity in the germinal center (GC) response. However, the degree of protection varies across the population, with older individuals often showing lower titer antibody responses to vaccines. Previous work has correlated increased exposure to Th1 cytokines, like IL‐2, with poor antibody responses in aging. Whether this is a causal relationship is unknown. Here we used *Il2* ^cre/+^; *Rosa26* ^stop‐flox‐Il2/+^ mice, which cannot shut down IL‐2 production once initiated, to test the hypothesis that sustained IL‐2 exposure affects the GC response and antibody production post vaccination. Prolonged IL‐2 production impeded T follicular helper (Tfh) cell differentiation, with reduced numbers of GC Tfh, pre‐Tfh and T follicular regulatory cells consistently observed in the draining lymph node of *Il2* ^cre/+^; *Rosa26* ^stop‐flox‐Il2/+^ mice at day 10 post immunization, relative to control mice. Numbers of Tfh cells remained suppressed at day 21 post immunization and corresponded with reduced GC B cell formation, including NP‐specific IgG1^+^ class‐switched GC B cells. As a core output of the GC response, B220^−^IRF4^+^CD138^+^ antibody‐secreting cells (ASCs) were significantly reduced in the bone marrow of *Il2* ^cre/+^; *Rosa26* ^stop‐flox‐Il2/+^ mice, 10 days post NP‐KLH immunization compared to control mice. However, by day 21, antigen‐specific humoral immunity was restored, with similar levels of B220^−^IRF4^+^CD138^+^ and NP‐specific ASCs in both experimental and control groups. In summary, while sustained IL‐2 production delayed Tfh differentiation and early GC formation, it did not affect overall antibody titers after vaccination.

## Graphical abstract

Sustained IL‐2 production delays T follicular helper cell differentiation and germinal center formation but does not alter final antibody titers or antigen‐specific humoral immunity after vaccination. Created in BioRender. Burton, A. (2026) https://BioRender.com/paggz41

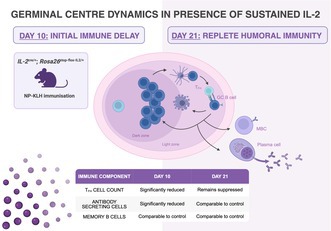

## INTRODUCTION

Vaccines have become a crucial tool in efforts to protect populations from infectious agents and eliminate diseases. Despite their success at the population level, vaccines do not protect all individuals equally. The causes of interindividual variation in response to immunization are diverse, with genetics thought to contribute minimally to vaccine success.^1^ However, both the age of the recipient and formulation of the vaccine play a significant role.^2^

The generation of protective humoral immunity following protein‐containing vaccines or infection occurs through the co‐ordination of CD4^+^ T follicular helper cells (Tfh) and antigen‐specific B cells, interacting together in secondary lymphoid organs at extrafollicular sites or within germinal centers (GCs). Successful collaboration within the GC leads to cyclical expansion and maturation of high‐affinity B cell clones, resulting in the output of highly specific, class‐switched antibody‐secreting cells (ASCs), as well as long‐lived memory B cells.^3^ Vaccine‐induced antibodies can also be generated via an extrafollicular pathway, by which cells bypass the GC response to produce a burst of short‐lived ASCs and memory B cells,^4^ supported by precursor Tfh (pre‐Tfh) cells acting outside of the B cell follicle.^5^ Aging is commonly associated with a persistent state of low‐grade inflammation, termed “inflammaging,” that is postulated to closely associate with immune impairment.^6^, ^7^, ^8^ In older people, where antibody production after vaccination is diminished, poor Tfh function is linked to an increase in circulating Tfh that have an inflammatory gene signature, including excess IL‐2 signaling.^9^, ^10^ We hypothesize that heightened inflammation may contribute to poorer responses to vaccination in older people,^11^ and that excess IL‐2 in particular may be detrimental to Tfh cell and GC formation.

IL‐2 is produced following early T cell activation and acts as a critical growth factor for the proliferation of T cells and generation of effector and memory cells. Signaling via IL‐2Rα (CD25) is central to coordinating T cell responses by promoting T regulatory cell (Treg) expansion while suppressing Tfh cell differentiation.^12^, ^13^, ^14^ Treatment with IL‐2, either as a monotherapy or in combination with other agents, has been used at high doses for many years to boost pro‐tumor responses in malignant disease,^15^ and at low doses to expand Treg populations and restore immune tolerance in autoimmune and inflammatory diseases.^16^ Through this balance, IL‐2 signaling likely plays a key role in regulating Tfh cell differentiation in inflammatory states.

Here, we used *Il2* ^cre/+^; *Rosa26* ^stop‐flox‐Il2/+^ mice that constitutively produce IL‐2 once expression has been initiated,^17^ to investigate the impact of sustained IL‐2 production on vaccine‐induced immune protection. We find that constitutive IL‐2 production significantly impacted the differentiation of all Tfh cell subsets studied after vaccination. However, the GC B cell and antibody response was able to recover in the absence of a complete Tfh compartment, with the abundance and affinity of antigen‐specific antibodies comparable between control and IL‐2‐overproducing mice. These data suggest that prolonged IL‐2 signaling is not sufficient to explain the diminished antibody responses in conditions of excess inflammation, such as aging.

## RESULTS

### Persistent IL‐2 production impairs early GC formation at day 10 post immunization

**Figure 1:**
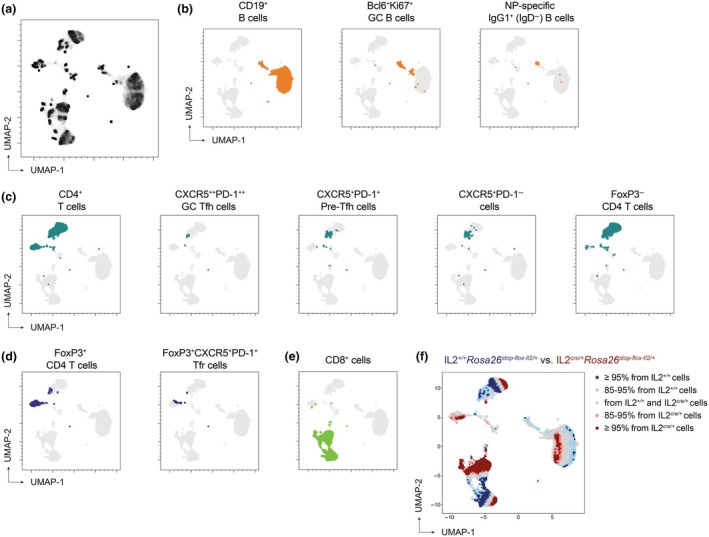
Sustained IL‐2 signaling alters the GC response to NP‐KLH immunization. T‐REX analysis of spectral flow data from lymphocytes isolated from the draining lymph node at day 21 post NP‐KLH immunization in *Il2* ^cre/+^; *Rosa26* ^stop‐flox‐Il2/+^ transgenic mice that do not switch off IL‐2 production, compared to *Il2* ^+/+^; *Rosa26* ^stop‐flox‐Il2/+^ control mice. **(a)** UMAP clustering was used to dimensionally reduce the data into lymphocyte subpopulations. Density of shading denotes the relative frequencies of cell types. Clusters include B cell populations (CD19^+^) **(b)**, which were subdivided into germinal center (GC) B cells (Bcl6^+^Ki67^+^) and NP‐specific IgG1^+^ (IgD^−^) B cells; FoxP3^−^ CD4^+^ T cell populations **(c)** which were subdivided into CXCR5^++^PD‐1^++^ GC T follicular helper cells (Tfh), CXCR5^+^PD‐1^+^ pre‐Tfh and CXCR5^+^PD‐1^−^ Tfh; **(d)** FoxP3^+^ T regulatory cells and CXCR5^+^PD‐1^+^ T follicular regulatory cells (Tfr); and **(e)** CD8^+^ T cells. **(f)** Lymphocyte clusters colored according to the relative contribution of each cell type from either *Il2* ^cre/+^; *Rosa26* ^stop‐flox‐Il2/+^ transgenic mice or *Il2* ^+/+^; *Rosa26* ^stop‐flox‐Il2/+^ control mice. Blue shading indicates cell types enriched in *Il2* ^+/+^; *Rosa26* ^stop‐flox‐Il2/+^ control mice, while red shading denotes cell types enriched in *Il2* ^cre/+^; *Rosa26* ^stop‐flox‐Il2/+^ transgenic mice.

To characterize the effect of persistent IL‐2 production on Tfh cell differentiation following immunization, *Il2* ^cre/+^; *Rosa26* ^stop‐flox‐Il2/+^ mice were immunized subcutaneously with NP‐KLH/Alhydrogel and GC responses in draining inguinal lymph nodes were analyzed by spectral flow cytometry. We began by dimensionally reducing the data using the machine learning algorithm Tracking Responders Expanding^18^ (T‐REX), to explore areas of difference between experimental *Il2* ^cre/+^; *Rosa26* ^stop‐flox‐Il2/+^ and control *Il2* ^+/+^; *Rosa26* ^stop‐flox‐Il2/+^ mice. This workflow first clustered the data using Uniform Manifold Approximation (UMAP) analyses (Figure 1a), revealing populations of GC B cells and NP‐specific IgG1^+^ B cells by 21 days post immunization (d.p.i; Figure 1b), along with Tfh cells (Figure 1c), Treg cells (CD4^+^FoxP3^+^) and T follicular regulatory (CD4^+^FoxP3^+^CXCR5^+^PD‐1^+^;Tfr) cells (Figure 1d), and CD8^+^ T cells (Figure 1e). T‐REX analysis was subsequently applied using a *k*‐nearest neighbors (KNN) search to identify UMAP clusters that differed between experimental and control mice. This analysis delineated regions of marked cellular expansion or contraction in *Il2* ^cre/+^; *Rosa26* ^stop‐flox‐Il2/+^ mice (Figure 1f), revealing that GC B cells and GC Tfh cells may be decreased in *Il2* ^cre/+^; *Rosa26* ^stop‐flox‐Il2/+^ mice. These findings were then further explored using manual gating across the time course of immunization (gating strategies in Supplementary figure 1).

**Figure 2:**
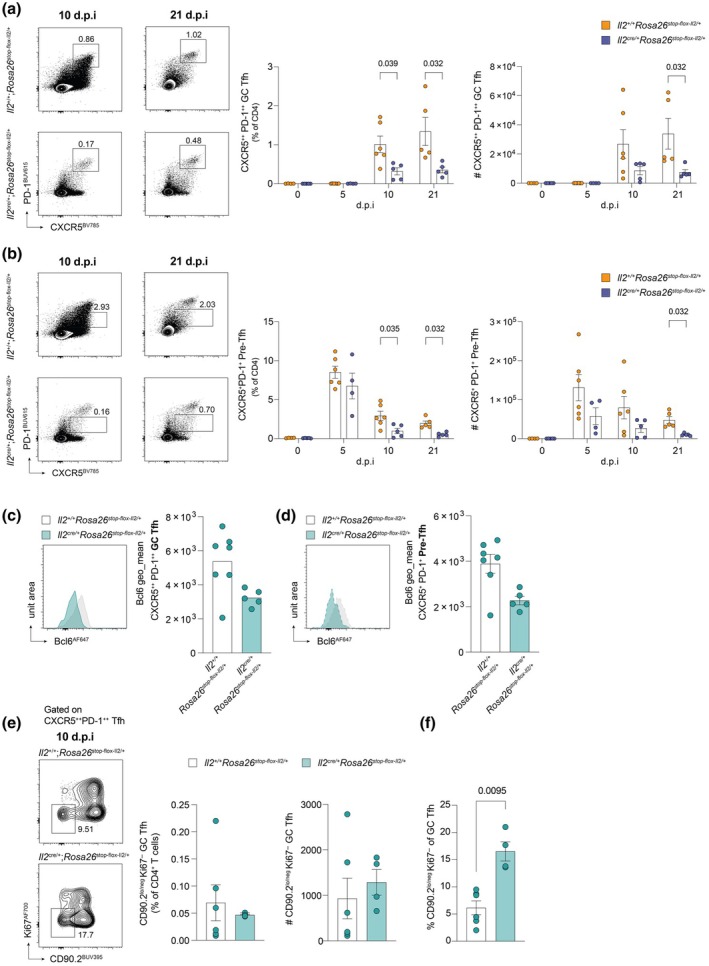
Increased IL‐2 production impairs T follicular helper differentiation following immunization. Assessment of Tfh cell subset frequency in the draining lymph node of *Il2* ^cre/+^; *Rosa26* ^stop‐flox‐Il2/+^transgenic mice, compared to *Il2* ^+/+^; *Rosa26* ^stop‐flox‐Il2/+^control mice post NP‐KLH immunization. **(a, b)** Representative flow cytometric plots and summary data showing the percentage and number of: **(a)** FoxP3^−^CXCR5^++^PD‐1^++^ germinal center Tfh (GC Tfh) and **(b)** FoxP3^−^CXCR5^+^PD‐1^+^ precursor Tfh (pre‐Tfh) in *IL2* ^+/+^; *Rosa26* ^stop‐flox‐Il2/+^ (orange) and *IL2* ^cre/+^; *Rosa26* ^stop‐flox‐Il2/+^ (purple) mice at 0, 5, 10 and 21 days post immunization (d.p.i). *n* numbers (control/experimental): 0 d.p.i = 4/5; 5 d.p.i = 6/4; 10 d.p.i = 6/5; 21 d.p.i = 5/5. **(c, d)** Expression of Bcl6 (geometric mean fluorescence intensity) in **(c)** FoxP3^−^CXCR5^++^PD‐1^++^ GC Tfh and **(d)** FoxP3^−^CXCR5^+^PD‐1^+^ pre‐Tfh at 10 d.p.i in *IL2* ^+/+^; *Rosa26* ^stop‐flox‐Il2/+^ (*n* = 7; gray) and *IL2* ^cre/+^; *Rosa26* ^stop‐flox‐Il2/+^ (*n* = 5; blue) mice. **(e, f)** Representative flow cytometric plots and summary data showing the percentage and number of CD90.2^low/negative^Ki67^−^ GC‐resident Tfh (as a percentage of total CD4^+^ T cells)_ **(e)** and the proportion of GC Tfh with a resident Tfh phenotype **(f)** in *IL2* ^+/+^; *Rosa26* ^stop‐flox‐Il2/+^ (*n* = 6; gray) and *IL2* ^cre/+^; *Rosa26* ^stop‐flox‐Il2/+^ (*n* = 4; blue) mice at 10 d.p.i. The full gating strategy is shown in Supplementary figure 1a, b. Data shown were obtained from one experiment per timepoint and are representative of at least two independent repeat experiments. Representative plots are taken from 10 and 21 d.p.i, unless otherwise stated. Error bars indicate mean ± s.e.m. *P*‐values were determined by multiple Mann–Whitney unpaired *t* tests.

NP‐KLH/Alhydrogel immunization induced robust GC Tfh cell (CXCR5^++^PD‐1^++^) formation by 10 d.p.i in control *Il2* ^+/+^; *Rosa26* ^stop‐flox‐Il2/+^ mice. When compared to mice unable to switch off IL‐2 production (*Il2* ^cre/+^; *Rosa26* ^stop‐flox‐Il2/+^ mice), Tfh cell frequencies were similar between groups at baseline (0 d.p.i) and 5 d.p.i (Figure 2a, b). However, at 10 d.p.i, there was a marked reduction in the proportion but not absolute number of GC Tfh and pre‐Tfh cells (CXCR5^+^PD‐1^+^) in *Il2* ^cre/+^; *Rosa26* ^stop‐flox‐Il2/+^ mice. Since IL‐2 has previously been shown to impede Tfh cell differentiation through the repression of the key transcription factor Bcl6,^12^ we compared the expression of Bcl6 in GC Tfh cells from control and experimental mice at 10 d.p.i by flow cytometry. Both GC Tfh and pre‐Tfh that were able to form in the draining lymph nodes of *Il2* ^cre/+^; *Rosa26* ^stop‐flox‐Il2/+^ mice tended to have lower expression of Bcl6 compared to those isolated from control mice (Figure 2c, d).

The frequency and number of GC Tfh and pre‐Tfh cells remained significantly decreased in mice that constitutively produced IL‐2 at 21 d.p.i, compared to controls (Figure 2a, b), indicating that sustained IL‐2 production inhibits Tfh cell differentiation in secondary lymphoid organs following vaccination, in line with previous reports.^12^, ^19^, ^20^

Sustained IL‐2 also inhibited the differentiation of CXCR5^+^PD‐1^−^ T helper cells at 21 d.p.i (Supplementary figure 2a, b), as has been shown in humans,^21^, ^22^ and transiently increased the frequency of Th1 cells at 10 but not 21 d.p.i (CXCR3^+^T‐bet^+^FoxP3^−^CD4^+^ T cells; Supplementary figure 2c, d). Interestingly, despite fewer Tfh cells overall, Tfh cells with a CD90.2^low^Ki67^−^ GC residency phenotype were proportionally enriched within the Tfh fraction of *Il2* ^cre/+^; *Rosa26* ^stop‐flox‐Il2/+^ experimental mice compared to control (Figure 2e, f). These data suggest that Tfh cells may become less sensitive to IL‐2 signaling once residency in the lymph node has been established, consistent also with observations showing decreased abundance of *IL2* transcripts in CD90.2^low^ lymph node resident Tfh.^23^

### Sustained IL‐2 production impairs T follicular regulatory cell differentiation

IL‐2 plays a critical role in balancing the immune system, promoting effector T cell responses while also providing immune control through the induction of *Foxp3* and maintenance of Treg differentiation in the thymus.^24^, ^25^ In the GC, specialized subsets of FoxP3^+^ Tfr cells use their suppressive function to limit the magnitude and output of the GC response. Hence, we next sought to investigate the impact of IL‐2 on the balance of Treg and Tfr cell abundance.

**Figure 3:**
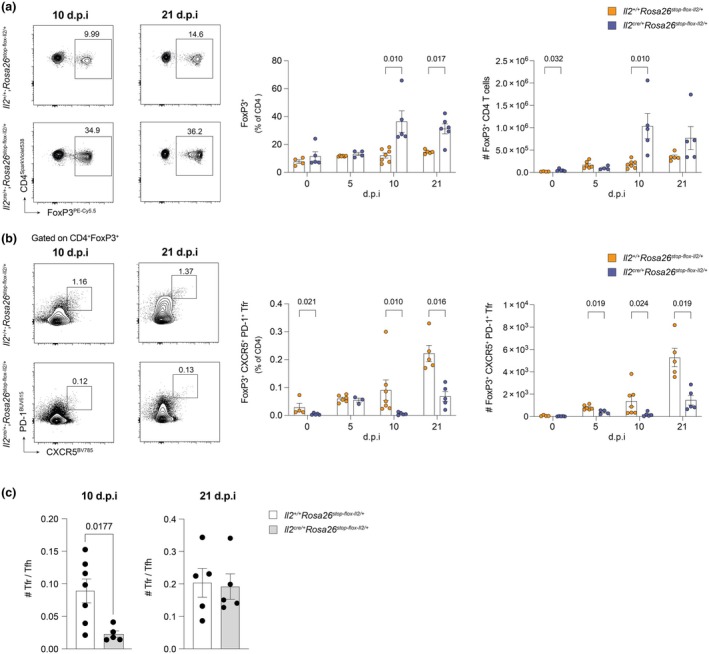
IL‐2 overproduction induces Treg but not Tfr expansion. Evaluation of T regulatory (Treg) and T follicular regulatory (Tfr) cell numbers in the draining lymph node of *Il2* ^cre/+^; *Rosa26* ^stop‐flox‐Il2/+^ transgenic mice, compared to *Il2* ^+/+^; *Rosa26* ^stop‐flox‐Il2/+^ control mice, post NP‐KLH immunization. **(a, b)** Representative flow cytometric plots and summary data showing the percentage and number of FoxP3^+^CD4^+^ Treg cells **(a)** and FoxP3^+^CD4^+^CXCR5^+^PD‐1^+^ Tfr cells **(b)** in *IL2* ^+/+^; *Rosa26* ^stop‐flox‐Il2/+^ (orange) and *IL2* ^cre/+^; *Rosa26* ^stop‐flox‐Il2/+^ (purple) mice at 0, 5, 10 and 21 days post immunization (d.p.i). *n* numbers (control/experimental): 0 d.p.i = 4/5; 5 d.p.i = 6/4; 10 d.p.i = 7/5; 21 d.p.i = 5/5. **(c)** Ratio of the number of Tfr to Tfh in *Il2* ^cre/+^; *Rosa26* ^stop‐flox‐Il2/+^ transgenic mice (gray) compared to *Il2* ^+/+^; *Rosa26* ^stop‐flox‐Il2/+^ control mice (white), at 10 and 21 d.p.i with NP‐KLH (*n* number (control/experimental) = 5/5). The full gating strategy is shown in Supplementary figure 1a, b. Data shown were obtained from one experiment per timepoint and are representative of at least two independent repeat experiments. Representative plots are taken from 10 and 21 d.p.i, unless otherwise stated. Error bars indicate mean ± s.e.m. *P*‐values were determined by multiple Mann–Whitney unpaired *t* tests **(a, b)** or the Mann–Whitney unpaired *t* test **(c)**.

Consistent with the role of IL‐2 in Treg expansion, IL‐2 overexpression increased the proportion of FoxP3^+^ CD4 Tregs, with a significantly higher proportion of Tregs seen in *Il2* ^cre/+^; *Rosa26* ^stop‐flox‐Il2/+^ mice at days 10 and 21 post immunization, compared to control mice (Figure 3a). Although Tfr cells share many overlapping phenotypic characteristics with Tregs, such as expression of GITR, CTLA‐4 and FoxP3,^26^, ^27^, ^28^, ^29^, ^30^ these cells lack expression of the high‐affinity receptor, IL‐2Rα (CD25): mature Tfr cells in the GC downregulate CD25 expression in comparison to Tregs and lose IL‐2 dependency, simultaneously gaining expression of Tfh‐related makers such as Bcl6 and CXCR5^31^ (Supplementary figure 3a–c). Therefore, we hypothesized that Tfr differentiation would be impaired in *Il2* ^cre/+^; *Rosa26* ^stop‐flox‐Il2/+^ mice, in line with repressed Tfh cell differentiation. As expected, the number and frequency of Tfr cells were decreased in *Il2* ^cre/+^; *Rosa26* ^stop‐flox‐Il2/+^ mice (Figure 3b), at 10 and 21 d.p.i compared to control mice. There was an initial reduction in the ratio of Tfr:Tfh cells (10 d.p.i) in *Il2* ^cre/+^; *Rosa26* ^stop‐flox‐Il2/+^ mice compared to control mice; however, this was resolved by 21 d.p.i (Figure 3c). Combined, these data support observations that chronic IL‐2 production promotes *Foxp3* expression and Treg frequency via signaling through IL‐2Rα, while mature Tfr cells are reduced in number.

### GC B cell responses are impaired in magnitude following vaccination in IL‐2 transgenic mice

**Figure 4:**
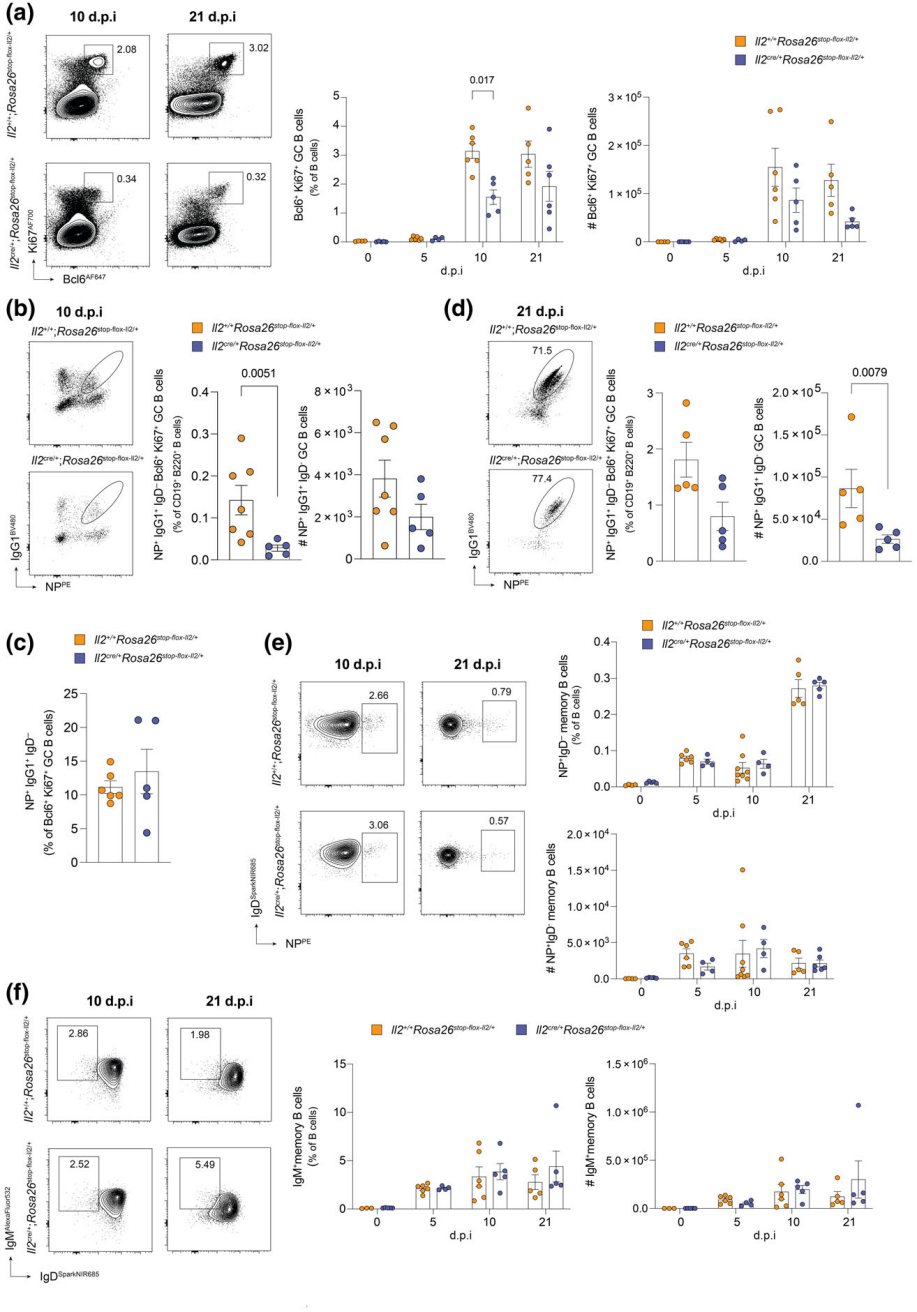
GC B cell responses are impaired in the presence of increased IL‐2 signaling. Assessment of germinal center (GC) and memory B cell frequency in the draining lymph node of *Il2* ^cre/+^; *Rosa26* ^stop‐flox‐Il2/+^ transgenic mice, compared to *Il2* ^+/+^; *Rosa26* ^stop‐flox‐Il2/+^ control mice, post NP‐KLH immunization. **(a)** Representative flow cytometric plots and summary data showing the percentage and number of Bcl6^+^Ki67^+^ GC B cells in *IL2* ^+/+^; *Rosa26* ^stop‐flox‐Il2/+^ (orange) and *IL2* ^cre/+^; *Rosa26* ^stop‐flox‐Il2/+^ (purple) mice at 0, 5, 10 and 21 days post immunization (d.p.i). *n* numbers (control/experimental): 0 d.p.i = 4/5; 5 d.p.i = 6/4; 10 d.p.i = 6/5; 21 d.p.i = 5/6. **(b)** Representative flow cytometric plots and summary data showing the percentage and number of NP^+^IgG1^+^ GC B cells in *IL2* ^+/+^; *Rosa26* ^stop‐flox‐Il2/+^ (*n* = 7; orange) and *IL2* ^cre/+^; *Rosa26* ^stop‐flox‐Il2/+^ (*n* = 5; purple) mice at 10 d.p.i. **(c)** Proportion of GC B cells that are NP‐specific and IgG1‐class‐switched between *IL2* ^+/+^ (*n* = 6; orange) and *IL2* ^cre/+^ (*n* = 5; purple) mice at 10 d.p.i. **(d)** Representative flow cytometric plots and summary data showing the percentage and number of NP^+^IgG1^+^ GC B cells in *IL2* ^+/+^; *Rosa26* ^stop‐flox‐Il2/+^ (*n* = 5; orange) and *IL2* ^cre/+^; *Rosa26* ^stop‐flox‐Il2/+^ (*n* = 5; purple) mice at 21 d.p.i. **(e)** Representative flow cytometric plots and summary data showing the percentage and number of NP‐specific IgD^−^Bcl6^−^Ki67^−^ memory B cells in *IL2* ^+/+^; *Rosa26* ^stop‐flox‐Il2/+^ (orange) and *IL2* ^cre/+^; *Rosa26* ^stop‐flox‐Il2/+^ (purple) mice at 0, 5, 10 and 21 d.p.i. *n* numbers (control/experimental): 0 d.p.i = 4/5; 5 d.p.i = 6/4; 10 d.p.i = 8/4; 21 d.p.i = 5/5. **(f)** Representative flow cytometric plots and summary data showing the percentage and number of IgM^+^IgD^−^Bcl6^−^Ki67^−^ memory B cells at 0, 5, 10 and 21 d.p.i. *n* numbers (control/experimental): 0 d.p.i = 3/5; 5 d.p.i = 6/4; 10 d.p.i = 6/5; 21 d.p.i = 5/5. The full gating strategy is shown in Supplementary figure 1a, c. Data shown were obtained from one experiment per timepoint and are representative of at least two independent repeat experiments. Representative plots are taken from the 10 and 21 d.p.i, unless otherwise stated. Error bars indicate mean ± s.e.m. *P*‐values were determined by multiple Mann–Whitney unpaired *t* tests.

Given the essential role of Tfh cells in the GC response, we hypothesized that reduced Tfh differentiation would lead to a corresponding decrease in the numbers of GC B cells. Frequencies of GC B cells were comparable between experimental and control mice, prior to immunization (Figure 4a). As anticipated, decreased Tfh cell differentiation was associated with lower frequencies of GC B cells at 10 d.p.i (Bcl6^+^Ki67^+^CD19^+^B220^+^ B cells; Figure 4a; Supplementary figure 1c), including NP‐ specific IgG1^+^ class‐switched GC B cells (Figure 4b), although absolute numbers of GC B cells were less impacted. However, the proportion of GC B cells that were class‐switched to IgG1 and NP‐specific at 10 d.p.i was comparable between *Il2* ^cre/+^; *Rosa26* ^stop‐flox‐Il2/+^ and control mice (Figure 4c), suggesting that constitutive IL‐2 production did not impair the induction of antigen‐specific immunity within the GC, but instead constrained the overall magnitude of the GC B cell response.

By day 21 post immunization, the number of global and NP‐specific IgG1^+^ GC B cells remained significantly lower in *Il2* ^cre/+^ *Rosa26* ^stop‐flox‐Il2/+^ mice compared to control *Il2* ^+/+^ *Rosa26* ^stop‐flox‐Il2/+^ mice (Figure 4a, d). These data led us to hypothesize that the downstream outputs of the GC – namely the formation of memory B cells, antigen‐specific ASCs and subsequent antibody production – might also be diminished in the presence of sustained IL‐2 production. Surprisingly, both the frequency and number of class‐switched memory B cells specific for NP, as well as IgM^+^ memory B cells, were comparable throughout the time course of immunization (Figure 4e, f). Thus, while constitutive IL‐2 production attenuated the magnitude of the GC response, it did not impair memory B cell formation.

### Generation of NP‐specific antibodies is not impacted by prolonged IL‐2 production

**Figure 5:**
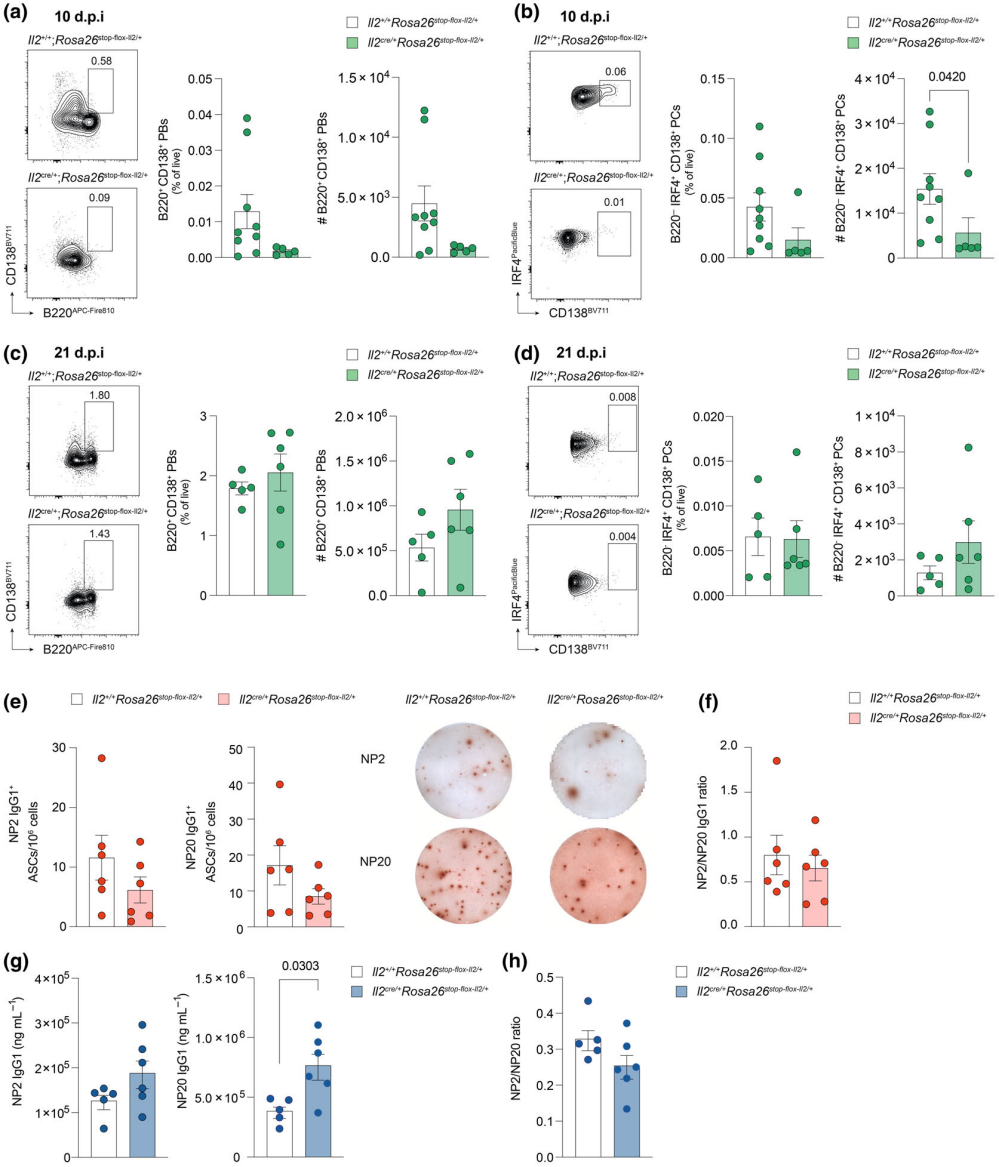
Vaccine‐specific humoral immunity is not impacted by IL‐2 overproduction. Assessment of the number and specificity of IgG1^+^ antibody secreting cells (ASCs) for NP2 or NP20 in the bone marrow of *Il2* ^cre/+^; *Rosa26* ^stop‐flox‐Il2/+^ transgenic mice, compared to *Il2* ^+/+^; *Rosa26* ^stop‐flox‐Il2/+^ control mice, post NP‐KLH immunization. **(a, b)** Representative flow cytometric plots and summary data showing the percentage and number of B220^+^CD138^+^ plasmablasts **(a)** and B220^−^CD138^+^IRF4^+^ plasma cells **(b)** in the bone marrow at 10 days post immunization (d.p.i) in *IL2* ^+/+^; *Rosa26* ^stop‐flox‐Il2/+^ (*n* = 9; white) and *IL2* ^cre/+^; *Rosa26* ^stop‐flox‐Il2/+^ (*n* = 5; green) mice. **(c, d)** Representative flow cytometric plots and summary data showing the percentage and number of B220^+^CD138^+^ plasmablasts **(c)** and B220^−^CD138^+^IRF4^+^ plasma cells **(d)** in the bone marrow at 21 d.p.i in *IL2* ^+/+^; *Rosa26* ^stop‐flox‐Il2/+^ (*n* = 5; white) and *IL2* ^cre/+^; *Rosa26* ^stop‐flox‐Il2/+^ (*n* = 6; green) mice. **(e)** Representative ELISPOT images and quantification of bone marrow NP2‐ and NP20‐specific IgG1^+^ ASCs in *IL2* ^+/+^; *Rosa26* ^stop‐flox‐Il2/+^ (*n* = 6; white) and *IL2* ^cre/+^; *Rosa26* ^stop‐flox‐Il2/+^ (*n* = 6; red) mice 21 d.p.i with NP‐KLH. **(f)** Affinity maturation of bone marrow ASCs at 21 d.p.i as determined by the ratio of NP2/NP20 specific ASCs. **(g)** Serum antibody titers of NP2‐ and NP20‐specific IgG1 in *IL2* ^+/+^; *Rosa26* ^stop‐flox‐Il2/+^ (*n* = 5; white) and *IL2* ^cre/+^; *Rosa26* ^stop‐flox‐Il2/+^ (*n* = 6; blue) mice 21 d.p.i with NP‐KLH. **(h)** Affinity maturation of antibodies at 21 d.p.i as determined by the ratio of NP2/NP20 IgG1 titers. Data shown were obtained from one independent experiment and are representative of two independent repeat experiments. Error bars indicate mean ± s.e.m. *P*‐values were determined by the Mann–Whitney unpaired *t* test **(a–e, g)** and the Mann–Whitney *t* test **(f, h)**.

To assess the impact of IL‐2 on the generation of humoral immunity, we quantified ASCs, defined as B220^−^CD138^+^IRF4^+^ plasma cells (PCs) and B220^+^CD138^+^ plasmablasts, in the bone marrow of experimental *Il2* ^cre/+^; *Rosa26* ^stop‐flox‐Il2/+^ and control *Il2* ^+/+^; *Rosa26* ^stop‐flox‐Il2/+^ mice, both prior to and post NP‐KLH immunization (gating strategy in Supplementary figure 1d). At baseline (0 d.p.i), frequencies of PCs and plasmablasts were comparable between experimental and control mice (Supplementary figure 4a, b), suggesting that persistent IL‐2 stimulation does not affect steady‐state humoral immune responses. Although not statistically significant, there was a notably lower proportion of plasmablasts and significantly fewer terminally differentiated PCs in the bone marrow of *Il2* ^cre/+^; *Rosa26* ^stop‐flox‐Il2/+^ compared to control mice at 10 d.p.i (Figure 5a, b), in line with a contracted GC response. However, by day 21 post immunization, the number and proportion of both plasmablasts and plasma cells in the bone marrow was restored to levels comparable to controls (Figure 5c, d), suggesting that ASC differentiation following immunization is maintained despite early defects in GC formation.

To further investigate the impact of IL‐2 on the generation of antigen‐specific immunity, we examined experimental and control mice at 21 d.p.i, at which point the quality and quantity of the humoral immune response could be assessed. NP‐specific ELISPOT assays were performed on bone‐marrow derived cells isolated from experimental and control mice at 21 d.p.i. In agreement with the flow data, there was no significant difference in the number of IgG1‐secreting ASCs specific for either NP2 or NP20 between experimental *Il2* ^cre/+^; *Rosa26* ^stop‐flox‐Il2/+^ and control mice (Figure 5e). Therefore, the ratio of high affinity NP2‐binding to NP20‐binding plasma cells was comparable between the experimental and control groups (Figure 5f), suggesting that affinity maturation and selection of vaccine‐specific clones is not impacted by constitutive IL‐2 production. Similarly, there was no difference in the concentration of NP2‐specific IgG1 measured in the serum at 21 d.p.i (Figure 5g), and while there were significantly higher levels of NP20‐specific IgG1 in *Il2* ^cre/+^; *Rosa26* ^stop‐flox‐Il2/+^ experimental mice (Figure 5g), the ratio of NP2‐IgG1/NP20‐IgG1 was consistent between conditions (Figure 5h). The frequency of plasmablasts (B220^+^CD138^+^) and B220^−^ plasma cells in the inguinal lymph nodes was comparable between groups at 5 d.p.i and thereafter (Supplementary figure 4c, d), suggesting that the extrafollicular B cell response was not impacted by constitutive IL‐2 production and may compensate where bone marrow responses are diminished. Similarly, titers of NP20 IgM at 10 and 21 d.p.i did not differ between experimental and control mice (Supplementary figure 4e, f). Together these data suggest that, despite early delays in Tfh and GC B cell differentiation, the magnitude and quality of humoral immunity output is maintained in mice that fail to silence IL‐2 production.

## DISCUSSION

Excessive IL‐2 has been identified as a central driver of Tfh cell dysfunction across many settings, including respiratory infection in early life^32^ and inflammatory liver disease,^33^ with defects in IL‐2 pathways potentially behind aberrant T helper cell responses common to many autoimmune conditions.^34^, ^35^ In light of evidence linking IL‐2 to Tfh cell suppression in aging, we sought to test the hypothesis that the persistent IL‐2 signaling seen in circulating Tfh‐like cells from older people^9^, ^10^ is causally linked to poor humoral immunity after vaccination. We used genetically modified mice where IL‐2 production cannot be inactivated once initiated and tested their response to vaccination. We confirm previous data showing that enhanced IL‐2 production limits Tfh cell differentiation and the immediate GC B cell response following NP‐KLH/Alhydrogel immunization, while simultaneously expanding populations of Tregs and Th1 cells. However, the overall quality and magnitude of the secretory antibody response was not impacted long‐term following overproduction of IL‐2, suggesting that aberrant IL‐2 signaling is not solely responsible for impaired antibody titers following vaccination in inflammatory settings such as aging.

IL‐2 activity has long been recognized as a key regulator of CD4 T cell fate determination.^36^, ^37^ In CD25‐expressing Tfh, IL‐2 signaling negatively modulates Bcl6 expression and represses Tfh differentiation through the activation of STAT5 and induction of BLIMP‐1 expression.^12^, ^19^ In contrast, IL‐2 signals favor the differentiation of other CD4 subsets, such as FoxP3^+^ Tregs in the peripheral blood^38^, ^39^ and Th1 effector cells.^37^ Our data confirm these observations, showing that enhanced IL‐2 production resulted in decreased numbers of Tfh cells following NP‐KLH/Alhydrogel immunization, compared to control mice. As anticipated, the number of Th1 and Tregs was increased in the presence of elevated IL‐2; however, as the key regulatory population in the GC, Tfr cells were decreased in *Il2* ^cre/+^; *Rosa26* ^stop‐flox‐Il2/+^ mice compared to controls due to their lack of expression of IL‐2Rα and dependency on Bcl6 expression.

Despite pronounced alterations to the CD4 T cell compartment upon persistent IL‐2 production, neither the induction of a humoral response following immunization, nor the affinity maturation of NP‐specific antibodies, was compromised. The maintenance of humoral immunity despite substantial suppression of Tfh and GC B cells implicates the involvement of additional pathways in antibody production. Indeed, the differentiation of extrafollicular plasmablasts and memory B cells in the draining lymph node remained largely unaffected by sustained IL‐2 signaling, indicating that these pathways may act in a compensatory capacity to sustain antibody production. In humans, IL‐2 is critical for commitment of naïve B cells to plasma cell fate via activation of ERK signaling^40^, ^41^ and can drive antibody production in already activated human B cells, even in the absence of Tfh cells.^33^ In mice, B cell‐intrinsic IL‐2 signaling induces expression of *Irf4* and *Prdm1*, which in turn promotes the energetic refueling required for ASC fate commitment in activated B cells and supports their differentiation into low‐mutated plasma cells at extrafollicular sites.^42^ Mice that received daily injections of recombinant IL‐2 showed significant increases in extrafollicular plasmablasts in place of GC B cells, resulting in substantial production of total and antigen‐specific IgG compared to controls.^42^ These data suggest that early exposure to IL‐2 during T‐B cell interactions can instruct ASC fate determination in IL‐2‐responsive B cells independently of the GC response, and may therefore explain how B cells remain able to differentiate into ASCs and produce comparable titers of immunoglobulin in mice that cannot turn off IL‐2 production, despite the collapse of the Tfh and GC B response. It may also support the finding that there are limits to the numbers of ASCs that can be maintained in the bone marrow niche, and that long‐lived plasma cells can differentiate via alternative pathways where the GC response is suppressed.^43^

In summary, our data reveal that the generation of protective humoral immunity following immunization is remarkably resilient to the impacts of sustained IL‐2 on the magnitude of the GC response. We previously showed that aged B cells preferentially enter extrafollicular pathways following immunization,^44^ suggesting that elevated IL‐2 levels with age may contribute to instructing plasma cell *versus* GC B cell fate commitment. However, elevated IL‐2 on its own does not explain the reduced antibody titers seen in older people. It is likely that multiple inflammatory pathways, such as TNF, underscore the association between inflammation and poor antibody titers, as previously observed.^9^, ^10^ Modulation of IL‐2 signaling for therapeutic purposes should be carefully considered due to its ability to modulate both CD4 T cell and B cell responses.

## METHODS

### Mouse maintenance and husbandry

*Il2* ^cre/+^; *Rosa26* ^stop‐flox‐Il2/+^ experimental and control *Il2* ^+/+^; *Rosa26* ^stop‐flox‐Il2/+17^ mice were bred and maintained at the Babraham Institute Biological Support Unit. Mice were kept in groups of 1–5 in individually ventilated cages, monitored daily and kept under pathogen‐free conditions, at an ambient temperature of 19–21°C with 52% relative humidity and fed CRM (P) VP diet (Special Diet Services) *ad libitum*.

### NP‐KLH/Alhydrogel immunizations

Mice were injected subcutaneously in both flanks with 100 μL of 50 μg NP‐KLH (#N‐5060‐25, Biosearch Technologies, Guildford, UK) in a 1:1 ratio with 2% (v/v) Alhydrogel (#vac‐alu‐50, Invivogen, Toulouse, France). Mice were immunized between 8 and 14 weeks of age. Male and female mice were used throughout. All research complied with relevant ethical regulation and was performed in the United Kingdom with approval from the Babraham Institute Animal Welfare and Ethical Review Body in compliance with European Union and UK Home Office legislation (Home Office License P4D4AF812 and PP9973990).

### Tissue processing and lymphocyte isolation

Single‐cell suspensions from inguinal lymph nodes were prepared by mechanically disrupting tissues through 40 μm cell strainers (#352340, Corning, Berlin, Germany) in FACs buffer (2%‐FBS (v/v) (#F9665, Sigma‐Aldrich, St Louis, MO, USA) in PBS with 0.05 mm EDTA (#15575020 Thermo Fisher Scientific, Waltham, MA, USA)). To isolate cells from bone marrow, femur bones were scraped clean and spun at 15,871 rcf/G for 1 min in a microcentrifuge. The resultant cell pellet was incubated with ammonium chloride buffer to lyse residual red blood cells for 5 min on ice, then washed with FACs buffer. Isolated cells were counted with CASY TT Cell Counter (OLS OMNI Life Science, Bremen, Germany).

### Flow cytometry

2 × 10^6^ cells were plated in a v‐bottom plate (#611 V96, Thermo Fisher Scientific) and stained with surface antibodies (Supplementary table 1) for 2 h at 4°C in FACs buffer. Cells were washed with FACs buffer and fixed with Foxp3/Transcription Factor Staining Buffer Set (#00–5323‐00, Invitrogen, Waltham, MA, USA) for 30 min at 4°C. NP‐specific cells were identified using a NP‐PE bait (#N‐5070‐B, Biosearch Technologies). Positive staining of NP was confirmed by comparison to negative cell populations (i.e. T cells) and unimmunized mice. For intracellular staining, cells were washed with FoxP3 Permeabilization Buffer (#00–8333‐56, Invitrogen) and stained with intracellular antibodies in FoxP3 Permeabilization Buffer at 4°C. 16 to 18 h later, cells were washed and resuspended in FoxP3 Permeabilization Buffer. Single color controls were created using spare splenocytes and stained as per samples. Data were acquired on a 5‐laser Cytek Aurora Spectral Analyzer (Cytek Biosciences Ltd. Ely, UK) and analyzed in FlowJo (v10.6.1, BD Biosciences, Franklin Lakes, NJ, USA). All antibodies used are listed in Supplementary table 1.

### Bone marrow enzyme‐linked immunosorbent spot assays (ELISpots)

ELISpots were performed as previously described.^45^ Briefly, multiscreen‐HA mixed cellulose ester plates (#MAHAS4510, Merck, Darmstadt, Germany) were coated with 10 μg mL^−1^ NP20‐BSA (#N‐5050H‐100, Biosearch Technologies) or 5 μg mL^−1^ NP2‐BSA (#N‐5050 L‐10, Biosearch Technologies) in PBS overnight at 4°C. Plates were blocked with DMEM (#41965–039, Gibco), supplemented with 10% (v/v) FBS (#F9665, Sigma), 1% (v/v) Penicillin–Streptomycin (#15140–122, Thermo Fisher Scientific) and 55 μM β‐mercaptoethanol (#21985023, Thermo Fisher Scientific) for 1 h at RT. Bone marrow cell suspensions were diluted down the plate in DMEM from a starting concentration of 2 × 10^6^ cells, and were incubated at 37°C, 5% CO_2_ overnight. Plates were then washed with 0.05% (v/v) Tween20 (#P1379, Sigma‐Aldrich) in PBS. Detection was performed with HRP‐conjugated anti‐mouse IgG1 (#ab97240, Abcam, Cambridge, UK) in 0.1% (w/v) BSA (#A7906, Sigma‐Aldrich), 0.05% (v/v) Tween20 (#P1379, Sigma‐Aldrich) in PBS. Plates were developed using the AEC staining kit (# AEC101, Sigma‐Aldrich). The number of ASCs was determined using a CTL ELISPOT reader (Cell Technologies, Cleveland, OH, USA) and the ImmunoSpot v.5.0 (Cellular Technology).

### ELISAs

For determination of anti‐NP antibody titers, flat‐bottom Nunc Maxisorp plates (#456537, Thermo Fisher Scientific) were coated with 10 μg mL^−1^ anti‐H + L IgG1 (#1030–01, Southern Biotech, Birmingham, USA), 10 μg mL^−1^ NP20‐BSA (#N‐5050H‐100, Biosearch Technologies) and 2 μg mL^−1^ NP2‐BSA (#N‐5050 L‐10, Biosearch Technologies) for 1 h at room temperature. Plates were washed 3 times in 0.05%‐Tween20 (v/v) (#P1379, Sigma‐Aldrich) in PBS using a Microplate Washer 405LS (Agilent BioTek, Winooski, Vermont, USA). All plates were blocked with 2% (w/v) BSA (#A7906, Sigma‐Aldrich) in PBS/Tween for 1 h at room temperature, followed by washing as described above. For the detection of NP‐specific IgG at 21 d.p.i, serum samples were initially diluted 1:1500 in 0.1% (w/v) BSA (#A7906, Sigma‐Aldrich) in PBS and plated in duplicate alongside duplicate wells of IgG isotype control at 150 μg mL^−1^ (#M5284, Sigma‐Aldrich). Both the IgG isotype control and serum samples were serially diluted down the plate at a 1:3 ratio.

Plates were washed and incubated with 0.000125 mg mL^−1^ HRP‐conjugated anti‐IgG1 antibody (#AB97420, Abcam) for 1 h at RT. For the detection of NP‐specific IgM, serum was instead incubated with 0.000125 mg mL^−1^ polyclonal HRP‐conjugated goat anti‐mouse‐IgM (#ab97230, Abcam) antibody for 1 h at RT. In all cases, plates were developed with TMB substrate set (#421101, BioLegend) and the reaction stopped with 2.5 m H_2_SO_4_. Absorbance was measured with PHERAstar FS plate reader (BMG Labtech, Ortenberg, Germany) at 450 nm.

### T‐REX analysis

T‐REX analysis was performed as published^18^ on concatenated live lymphocyte events from mice 21 d.p.i with NP‐KLH (25 000 cells/mouse from 5 experimental *Il2* ^cre/+^; *Rosa26* ^stop‐flox‐Il2/+^ and 6 control *Il2* ^+/+^; *Rosa26* ^stop‐flox‐Il2/+^ mice) using the full spectral panel outlined in Supplementary table 1. QC of spectral data were performed prior to analysis, including spectral unmixing, autofluorescence correction and manual gating to exclude debris, doublets and dead cells. 25,000 live lymphocyte events were downsampled from each mouse and concatenated. UMAP analyses were run using standard parameters (perplexity, 30; iterations, 1000; minimum cluster size, 10). UMAP parameters were selected based on their ability to reproducibly identify the expected populations of interest across multiple independent runs. The T‐REX algorithm was applied using a *K*‐value of 60 and epsilon of 0.30. All analyses were performed using the T‐REX plugin in FlowJo (v10.6.1).

### Statistical analysis

Statistical analyses were performed in Prism 10 (GraphPad Software, Boston, MA, USA) using appropriate methods as indicated in the legends, with all *P*‐values marked on figures. All tests were performed as two‐tailed tests.

## CONFLICT OF INTEREST

MAL reports funding from GSK outside of this work. AL is founder of Aila Biotech Ltd. All other authors declare no competing financial interests.

## AUTHOR CONTRIBUTIONS

**Silvia Innocentin:** Methodology; investigation; sample provision; writing – original draft; writing – review and editing. **Ross McKenzie:** Methodology; investigation; writing – original draft; writing – review and editing. **Jayalini Assalaarachchi:** Methodology; investigation; sample provision; writing – review and editing. **Helena A Carslaw:** Methodology; investigation; sample provision; writing – review and editing. **Anusha Gupta:** Methodology; investigation; writing – review and editing. **Sanne Cole:** Investigation; writing – review and editing. **Adrian Liston:** Supervision; sample provision; writing – review and editing. **Michelle A Linterman:** Conceptualisation; funding acquisition; supervision; methodology; writing – original draft; writing – review and editing. **Louise Webb:** Methodology; supervision; investigation; writing – review and editing. **Alice R Burton:** Conceptualisation; funding acquisition; methodology; investigation; sample provision; supervision; writing – original draft; writing – review and editing.

## Supporting information


Supplementary data 1

## Data Availability

The data that support the findings of this study are available from the corresponding author upon reasonable request.
