# Supplementary material for: IL‐2 silencing enables Tfh cell expansion during vaccination but is redundant for antibody production
Source: Immunol Cell Biol. 2026 Feb 24;104(4):316–28. doi: 10.1111/imcb.70084 (PMC13071127; doi:10.1111/imcb.70084)
Supplement: Supplementary file 1 — Supplementary data 1 [file IMCB-104-316-s001.pdf]

Supplementary figure 1. Full gating strategy for identification of lymphocyte subsets

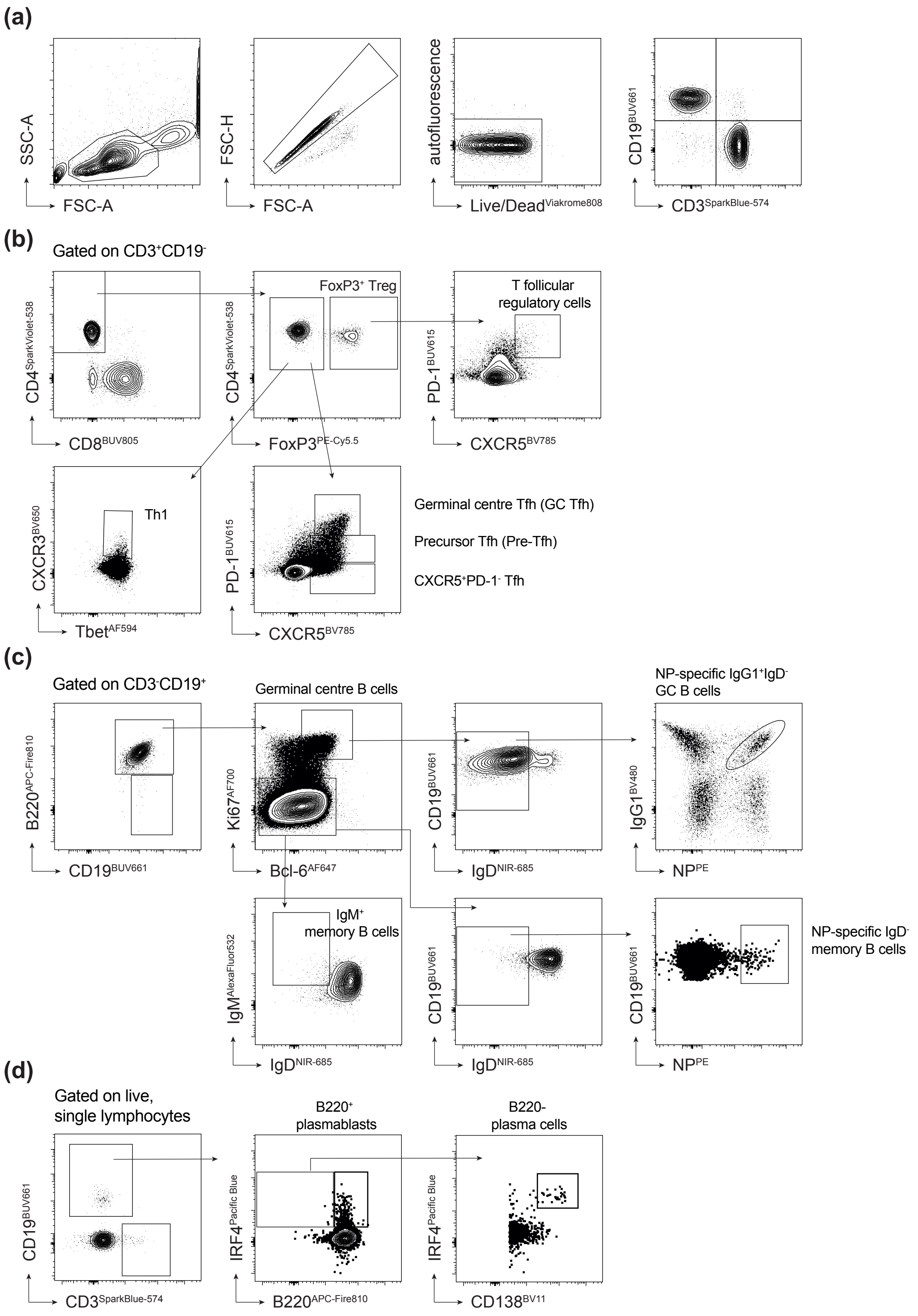

Supplementary figure 1: Full gating strategies for the identification of lymphocyte subsets

Flow cytometric gating strategy defining lymphocyte subsets in the draining lymph nodes and bone marrow of immunised mice. **(a)** Live lymphocytes defined through sequential exclusion of debris and mononuclear phagocytes by forward (FSC-A) and side scatter (SSC-A); doublets; dead and auto-fluorescent cells. **(b-d)** Sequential gating strategies to define CD4 T cell subsets **(b)**, B cell subsets **(c)**, plasmablasts and plasma cells **(d)**.

Supplementary figure 2. IL-2 signaling regulates CD4 T cell fate following immunisation

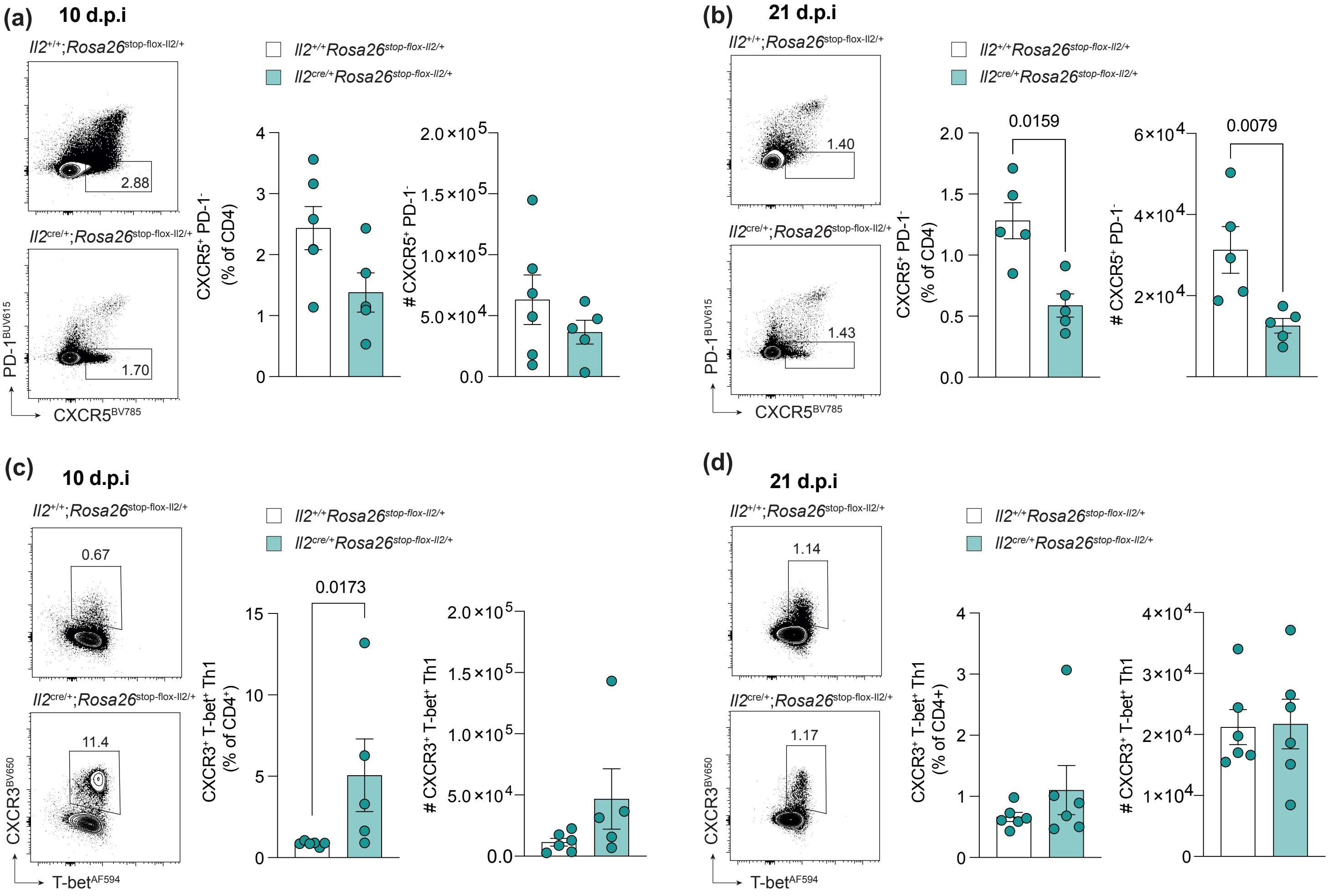

Supplementary figure 2: IL-2 signaling regulates CD4 T cell fate following immunisation

Evaluation of CXCR5$^+$PD-1$^-$ Tfh and T helper 1 (Th1) differentiation in the draining lymph node at day 10 and day 21 post NP-KLH immunisation. **(a-b)** Representative flow cytometric plots and summary data showing the percentage and number of FoxP3$^-$CXCR5$^+$PD-1$^-$ CD4$^+$ T cells in IL2$^{+/+}$; Rosa26$^{stop\text{-}flox\text{-}Il2/+}$ (n = 5) and IL2$^{cre/+}$; Rosa26$^{stop\text{-}flox\text{-}Il2/+}$ (n = 5) mice at 10 days **(a)** and 21 days **(b)** post immunisation (d.p.i). **(c-d)** Representative flow cytometric plots and summary data showing the percentage and number of FoxP3$^-$CXCR3$^+$T-bet$^+$ Th1 CD4$^+$ T cells in IL2$^{+/+}$; Rosa26$^{stop\text{-}flox\text{-}Il2/+}$ (n = 6) and IL2$^{cre/+}$; Rosa26$^{stop\text{-}flox\text{-}Il2/+}$ (n = 5) mice at 10 days **(c)** and 21 days **(d)** post immunisation (d.p.i). Data is representative of two independent experiments. Error bars indicate mean ± SEM. *P*-values were determined by the Mann-Whitney unpaired *t* test.

Supplementary figure 3. Mature Tfr are not dependent on IL-2 signaling

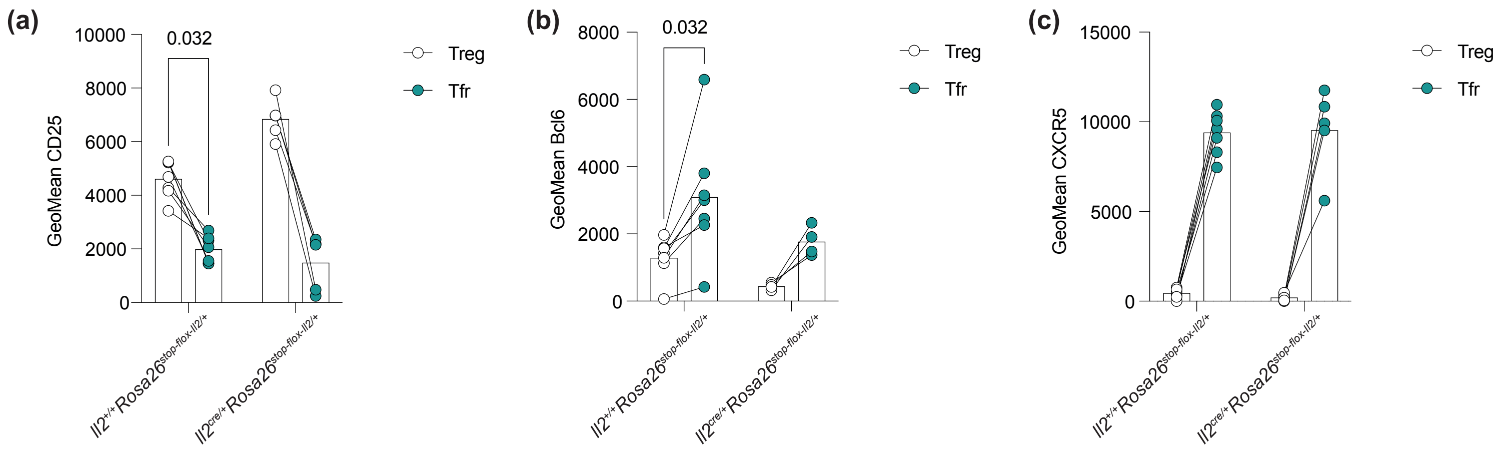

Supplementary figure 3: Mature Tfr are not dependent on IL-2 signaling

(a-c) Summary data showing the expression (geometric mean fluorescence intensity) of CD25 (IL-2R; **a**), Bcl6 **(b)** and CXCR5 **(c)** on FoxP3$^+$CD4$^+$ Tregs or FoxP3$^+$CXCR5$^+$PD-1$^+$ Tfr in IL2$^{+/+}$; Rosa26$^{stop\text{-}flox\text{-}Il2/+}$ (n = 7) and IL2$^{cre/+}$; Rosa26$^{stop\text{-}flox\text{-}Il2/+}$ (n = 5) mice at day 10 post NP-KLH immunisation. The full gating strategy is shown in Supplementary figure 1a, b. Data is representative of two independent experiments. Error bars indicate mean ± SEM. *P*-values were determined multiple Wilcoxon tests.

Supplementary figure 4. Extrafollicular B cell differentiation is not altered by persistent IL-2 signaling

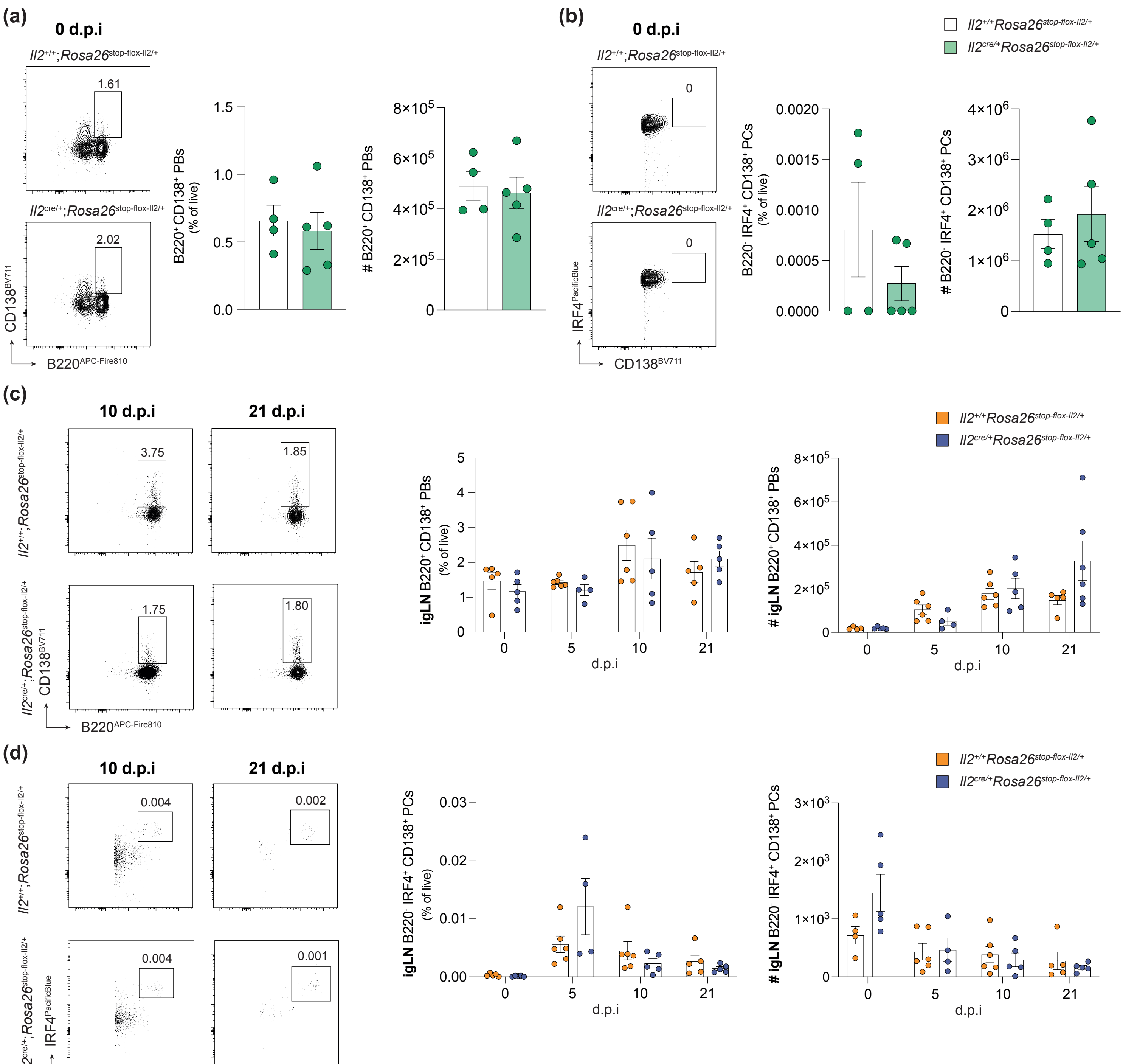

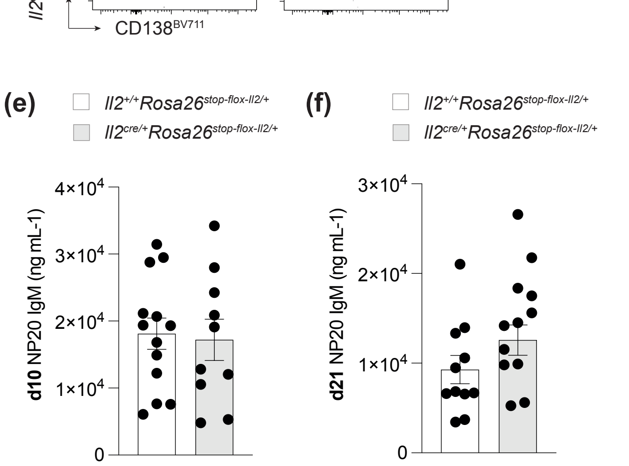

Supplementary figure 4: Extrafollicular B cell differentiation is not altered by persistent IL-2 signaling

Assessment of plasmablasts and plasma cells prior to and following NP-KLH immunisation. **(a-b)** Representative flow cytometric plots and summary data showing the percentage and number of B220$^+$CD138$^+$ plasmablasts **(a)** and B220$^-$CD138$^+$IRF4$^+$ plasma cells **(b)** in the bone marrow prior to NP-KLH immunisation (0 d.p.i) in IL2$^{+/+}$; Rosa26$^{stop\text{-}flox\text{-}Il2/+}$ (n = 4) and IL2$^{cre/+}$; Rosa26$^{stop\text{-}flox\text{-}Il2/+}$ (n = 5) mice. **(c-d)** Representative flow cytometric plots and summary data showing the percentage and number of B220$^+$CD138$^+$ plasmablasts **(c)** and B220$^-$CD138$^+$IRF4$^+$ plasma cells **(d)** in the inguinal lymph nodes at 0, 5, 10 and 21 days post immunisation (d.p.i). n numbers (control/experimental): 0 d.p.i = 5/5; 5 d.p.i = 6/4; 10 d.p.i = 6/5; 21 d.p.i = 5/5. **(e)** Serum antibody titres of NP20-specific IgM in IL2$^{+/+}$; Rosa26$^{stop\text{-}flox\text{-}Il2/+}$ (n = 13) and IL2$^{cre/+}$; Rosa26$^{stop\text{-}flox\text{-}Il2/+}$ (n =10) mice at day 10 post NP-KLH immunisation. **(f)** Serum antibody titres of NP20-specific IgM in IL2$^{+/+}$; Rosa26$^{stop\text{-}flox\text{-}Il2/+}$ (n = 11) and IL2$^{cre/+}$; Rosa26$^{stop\text{-}flox\text{-}Il2/+}$ (n = 12) mice at day 21 post NP-KLH immunisation. The full gating strategy is shown in Supplementary figure 1a, d. Data is representative of at least two independent experiments. Representative plots are taken from the peak (10 d.p.i) and resolution (21 d.p.i) of the GC response, unless otherwise stated. Error bars indicate mean ± SEM. *P*-values were determined by multiple Mann-Whitney unpaired *t* tests.

Supplementary table 1: Full list of antibodies used for flow cytometric analysis

**Fluorophore**	**Target**	**Clone**	**Company**	**Cat. #**
BUV395	CD90	53-2.1	BD Biosciences	565257
BUV496	CD23	B3B4	BD Biosciences	741058
BUV615	PD1	RMP130	BD Biosciences	752354
BUV661	CD19	1D3	BD Biosciences	612971
BUV737	CD62L	MEL-14	BD Biosciences	612833
BUV805	CD8a	53-6.7	BD Biosciences	612898
PacBlue	IRF4	IRF4.3E4	Biolegend	646418
BV480	IgG1	A85-1	BD Biosciences	746811
BV510	CD35/21	7G6	Biolegend	747764
SV538	CD4	GK1.5	Biolegend	100485
BV605	CD86	GL-1	Biolegend	105037
BV650	CXCR3	CXCR3-173	Biolegend	126531
BV711	CD138	281-2	Biolegend	142519
BV750	ICOS	c398.4a	Biolegend	313558
BV785	CXCR5	L138D7	Biolegend	145523
Alexa 532	IgM	II/41 (A20182)	Invitrogen (inhouse conjugate)	14-5790-82 A20182 Kit
SparkBlue574	CD3	17A2	Biolegend	100275
BB700	GATA3	L50-823	BD Biosciences	566642
PE	NP		LGC Biosearch	N-5070-1
SparkYG593	CD11b	M1/70	Biolegend	B368393
AF594	T-bet	4B10	Biolegend	644834
PE-dazzle594	CCR6	29-2L17	Biolegend	129822
PE-Fire700	CD38	90	Biolegend	102747
PE-Cy5	CD69	H1.2F3	Biolegend	104510
PE-Cy5.5	Foxp3	FJK-16S	Invitrogen	35-5773-82
PE-Cy7	GL7	GL7	Biolegend	144620
APC	CXCR4	I.276F12	Biolegend	146508
Alexa 647	Bcl-6	IG191E/A8	Biolegend	648306
SparkNIR685	IgD	11-26c.2a	Biolegend	405750
Alexa 700	Ki67	SolA1	Biolegend	56-5698-82
Viakrome808	viability		BeckmanCoulter	C36628
APC-eFluor780	CD25	PC61.5	eBioscience	47-0251-82
APCFire810	B220	RA3-6B2	Biolegend	103278
